# On Brand Stereotyping: Conceptual Specifications and Further Insight

**DOI:** 10.3389/fpsyg.2022.867667

**Published:** 2022-05-13

**Authors:** Georgios Halkias

**Affiliations:** Copenhagen Business School, Frederiksberg, Denmark

**Keywords:** stereotypes (social psychology), branding (marketing), consumer–brand relationship, categorization, market knowledge

## Introduction

Treating brands as algebraic combinations of discrete features is arguably myopic, as it neglects that brands represent social entities firmly embedded in people's environment and social interactions (Arnould and Thompson, 2005). Indeed, brands' societal role is in the spotlight of the marketing literature with scholars over the recent years drawing heavily from stereotype theory in social psychology, and particularly the Stereotype Content Model (SCM; Fiske et al., 2002), to study how consumers think about, feel for, and connect with brands. The transition from social to brand stereotyping is a challenging new endeavor with a notably growing number of studies subscribing to it (Davvetas and Halkias, 2019; Halkias and Diamantopoulos, 2020). However, while the number of relevant empirical applications is spiraling, discussions about the conceptual specifications and the compatibility of these two domains are largely being neglected. As a result, the current state of theorizing is underdeveloped and empirical findings on brand stereotyping draw from diverse and often tentative theorizing. As MacInnis (2012) argues in her commentary to Kervyn's et al. (2012) seminal paper on brand stereotypes, before the potential value of a stereotyping framework can be assessed, greater theoretical detailing is necessary. Failure to do so, hinders the identification of important phenomena based on brands' societal status. This paper acts as a primer bringing forward considerations which stimulate debate and inform further explorations in the dynamic and growing literature on brand stereotypes.

## Capturing Stereotypes

The SCM is one of the most widely used frameworks to investigate stereotyping and draws on the idea that individuals' social perception is largely determined by two fundamental dimensions, namely *warmth* and *competence* (Fiske et al., 2002). These dimensions are based on evolutionary theory and suggest that when people encounter “others” they intuitively want to know *(a)* whether “others” have favorable or unfavorable intents toward them (warmth) and *(b)* whether “others” can effectively pursue their intents (competence) (Fiske et al., 2002). Social groups with more cooperative (competitive) intentions, such as the elderly (ghetto people), are primarily perceived as warm (cold), whereas groups that have (lack) the power to implement their intentions, such as the Wall Street professionals (homeless people), are primarily perceived as competent (incompetent). Judgments along these dimensions are found to shape behavioral tendencies toward these groups and its members (Fiske et al., 2002). The efficacy of the SCM in predicting behavior has been supported in several contexts of social perception and at various degrees of specificity, ranging from whole cultures and entire countries to social groups and individual persons (Halkias and Diamantopoulos, 2020).

The elegant and versatile nature of the SCM has also given rise to a novel research paradigm i.e., *brand stereotyping*. Landmark in this endeavor, was a recent Research Dialogue in the *Journal of Consumer Psychology* (Vol. 22, Issue 2, 2012) in which Kervyn et al. (2012) integrated theories from social cognition and consumer–brand relationships in an attempt to bridge social and brand perception. More specifically, Kervyn et al. (2012) proposed the *Brands as Intentional Agents Framework* (BIAF) as an adaptation of the SCM to fit the brand domain. The BIAF suggests that consumer–brand interactions are also driven by the two SCM dimensions, which Kervyn et al. (2012) referred to as *intention* and *ability*. According to the BIAF, brands are seen as agents that carry intentionality and act in a purposeful manner. As such, they may be perceived as having cooperative and altruistic or competitive and exploitative intentions (brand intent/warmth) and, at the same time, they may be perceived as either possessing or lacking the ability to enact upon these intentions (brand ability/competence) (Kervyn et al., 2012).

The BIAF and the general idea that stereotype theory can be applied to study the perception of brands sparked particular interest with an increasing number of studies drawing on perceptions of brand warmth and brand competence (Ivens et al., 2015; Halkias and Diamantopoulos, 2020). However, extant empirical applications are largely uncritical, or even superficial, in terms of the conceptual specifications required to apply stereotyping theory for the perceptions of products and brands (cf. Davvetas and Halkias, 2019). Studies often jump to the empirical operationalization of brand stereotypes (i.e., measurement of brand warmth/competence), as if a complete and self-evident compatibility between social stereotyping and brand stereotyping exists. We argue that this is hardly the case and that important conceptual issues have been overlooked, inevitably casting doubt on the validity of the relevant findings.

Is the transition from social to brand stereotyping axiomatically established? Can stereotype theory, which was essentially developed to account for how societies view people belonging to certain social groups, readily accommodate the perception of non-human entities such as brands? We would argue that it can, so long as the common conceptual denominator between these two domains is identified. And, to do so, one should bear in mind that stereotype theory deals with the perception of *human agents* that are *members of a particular social group* (McGarty et al., 2002). A valid research paradigm has to account for both of these elements. In what follows, I offer some conceptual insight to this direction.

## Brands as Social Entities

Brands have a long-standing and impactful presence in the human society. People grow up with brands which, oftentimes, accompany them throughout their entire lives. In fact, many successful brands live longer than people do; they have been inherited from previous generations and will likely be passed on to the ones that follow. Take, for instance, *Coca-Cola* which is long past its 125th birthday. Similarly, *Colgate* first introduced its packaged toothpaste as early as 1873, while the famous *Levi's* logo was originally patched onto the company's jeans in 1,886 and is still used today. In addition, people get attached to, develop emotions for, and identify with brands (Langner et al., 2016). *Harley-Davidson* owners and *Apple* users are typical examples of such special consumer–brand connections. Finally, people tend to anthropomorphize brands (Eisend and Stokburger-Sauer, 2013) and, acknowledging this, companies nowadays explicitly ascribe humanlike characteristics to brands as, for example, *Amazon*'s *Alexa* and *Apple*'s *Siri*. Overall, there is already ample evidence to suggest that people can relate to brands quite similarly to the way they relate to people around them (Fournier and Alvarez, 2012). Thus, it is reasonable to think of brands as inanimate entities of our social reality that do carry some form of humanlike agency.

## Brands as Group Members

Whether a brand will actually be stereotyped, however, also depends on group membership. A stereotype is a commonly held set of beliefs about the characteristics of a particular social group or category, while stereotyping is the process of assigning category characteristics to individual members simply because of their membership to a given category (McGarty et al., 2002). Therefore, stereotyping is an inevitable consequence of categorization. Accordingly, a brand may be subjected to stereotyping so long as membership to a distinct category or group can be established.

Marketing literature suggests that consumers organize market knowledge in the form of superordinate and subordinate cognitive schemata, ranging from generic product classes (e.g., *beverages*) to distinct product categories (e.g., *soft-drinks*), more refined subordinate product categories (e.g., *sodas*), and specific brands (e.g., *Dr. Pepper*) (Halkias, 2015). Unlike such “functional” hierarchical structures, consumers often organize products and brands in thematic chunks corresponding to distinct groups or categories for which people hold purchase-relevant stereotypical beliefs. For instance, consumers intuitively categorize products in generic categories such as *value-for-money, luxury, upcycled, private-label, eco-friendly, nonprofit, local, global*, or *handmade*, to name but a few. To the extent that a brand possesses diagnostic enough features (e.g., price, country of origin), activation of a distinct such category can be triggered (e.g., luxury brands, local brands) and the process of brand stereotyping can take place. Surprisingly, with very few exceptions (see Aaker et al., 2010; Davvetas and Halkias, 2019), the notion of brand group membership has been conceptually and empirically ignored in extant research.

## Brand Stereotyping Process

Categorization and stereotyping typically occurs as individuals go through the available features of a stimulus target. During this process, a number of features diagnostic of a social group might be identified, thus enabling categorization of the individual stimulus to the category activated. For example, imagine you encounter a male person with straight, black hair, little facial hair, and dark, wide eyes. Unlike the rest of the features, “wide eyes” is naturally diagnostic to activate the category “Asians” and ascribe membership of the particular individual therein. Thus, given the stereotype that Asians are good at mathematics, you would tend to believe that the person encountered also possesses this quality. Brand stereotyping should occur in a similar way (Davvetas and Halkias, 2019). Salient product attributes can function as diagnostic cues that activate superordinate groups of products or brands which, in turn, are characterized by specific stereotypical properties. Once a brand is assigned membership to such a category, category properties will transfer to the perception of the individual product. In the context of the SCM/BIAF model, stereotypical brand judgements can be formed as some dominant brand feature (e.g., *worldwide availability*) instigates a superordinate category (e.g., *global brands*) associated with a particular stereotype content configuration (e.g., *more competent than warm*) which, in turn, transfers to the perception of the individual brand (Davvetas and Halkias, 2019). Similar processes may very well exist for other market-relevant classifications.

## Summary

This piece discusses the notion of brand stereotyping in the hope of stimulating debate and further theoretical refinement in the dynamic and growing literature of brand stereotypes. Brands represent social elements which consumers mentally organize into superordinate collective entities or categories that vary in how well-intentioned (warmth) and how able (competence) they are generally perceived to be. Brand stereotyping is contingent on assigning an individual brand to one such categories. Once brand membership is established, category beliefs will influence how the individual brand is perceived, having downstream influences on behavioral responses. Importantly, and unlike what most existing empirical studies imply, stereotypical judgments of warmth and competence about a specific brand are indirect, driven by how the category which the brand is assigned to is stereotyped.

Researchers are encouraged to critically reflect on the propositions above and contribute to the development of a unified robust research paradigm about brand stereotyping. Experimental studies in consumer psychology should employ creative designs to empirically establish brand categorization processes and explore any causal linkages between brand category perceptions, individual brand judgments, and subsequent consumer responses. In this context, particular emphasis should be placed on identifying key stereotypical (brand) categories (e.g., *private-label, handmade, upcycled, repurposed, born-global*, and *AI-made* brands) as well as diagnostic/prototypical features that may instigate brand stereotyping. Interpretive-driven qualitative studies, drawing on case studies, ethnography, action research, and grounded theory can be particularly valuable to this direction. Finally, cross-cultural studies should explore whether the emerging brand categories are stereotyped in a uniform way across cultural contexts and whether brands' stereotype content is generalizable beyond single-country contexts.

## Author Contributions

The author confirms being the sole contributor of this work and has approved it for publication.

## Conflict of Interest

The author declares that the research was conducted in the absence of any commercial or financial relationships that could be construed as a potential conflict of interest.

## Publisher's Note

All claims expressed in this article are solely those of the authors and do not necessarily represent those of their affiliated organizations, or those of the publisher, the editors and the reviewers. Any product that may be evaluated in this article, or claim that may be made by its manufacturer, is not guaranteed or endorsed by the publisher.

## References

[B1] Aaker J. Vohs K. D. Mogilner C. (2010). Nonprofits are seen as warm and for-profits as competent: firm stereotypes matter. J. Consum. Res. 37, 224–237. 10.1086/651566

[B2] Arnould E. J. Thompson C. J. (2005). Consumer Culture Theory (CCT): Twenty years of research. J. Consum. Res. 31, 868–882. 10.1086/426626

[B3] Davvetas V. Halkias G. (2019). Global and local brand stereotypes: formation, content transfer, and impact. Int. Market. Rev. 36, 675–701. 10.1108/IMR-01-2018-0017

[B4] Eisend M. Stokburger-Sauer N. E. (2013). Brand personality: a meta-analytic review of antecedents and consequences. Mark. Lett. 24, 205–216. 10.1007/s11002-013-9232-7

[B5] Fiske S. T. Cuddy A. J. C. Glick P. Xu J. (2002). A model of (often mixed) stereotype content: competence and warmth respectively follow from perceived status and competition. J. Person. Soc. Psychol. 82, 878–902. 10.1037/0022-3514.82.6.878 12051578

[B6] Fournier S. Alvarez C. (2012). Brands as relationship partners: warmth, competence, and in-between. J. Consum. Psychol. 22, 177–185. 10.1016/j.jcps.2011.10.003

[B7] Halkias G. (2015). Mental representation of brands: a schema-based approach to consumers' organization of market knowledge. J. Product Brand Manag. 24, 438–448. 10.1108/JPBM-02-2015-0818

[B8] Halkias G. Diamantopoulos A. (2020). Universal dimensions of individuals' perception: Revisiting the operationalization of warmth and competence with a mixed-method approach. Int. J. Res. Market. 37, 714–736. 10.1016/j.ijresmar.2020.02.004

[B9] Ivens B. S. Leisching A. Muller B. Valta K. (2015). On the role of brand stereotypes in shaping consumer response toward brands: an empirical examination of direct and mediating effects of warmth and competence. Psychol. Market. 32, 808–820. 10.1002/mar.20820

[B10] Kervyn N. Fiske S. T. Malone C. (2012). Brands as intentional agents framework: how perceived intentions and ability can map brand perception. J. Consum. Psychol. 22, 166–176. 10.1016/j.jcps.2011.09.006 24403815 PMC3882007

[B11] Langner T. Bruns D. Fischer A. Rossiter J. R. (2016). Falling in love with brands: a dynamic analysis of the trajectories of brand love. Mark. Lett. 27, 15–26. 10.1007/s11002-014-9283-4

[B12] MacInnis D. J. (2012). Brands as intentional agents: questions and extensions. J. Consum. Psychol. 22, 195–198. 10.1016/j.jcps.2011.10.004 PMC3882007 24403815

[B13] McGarty C. Yzerbyt V. Y. Spears R. (2002). Stereotypes as Explanations: The Formation of Meaningful Beliefs About Social Groups. Cambridge: University Press.

